# Supplementary material for: Left and Right Cortical Activity Arising from Preferred Walking Speed in Older Adults
Source: Sensors (Basel). 2023 Apr 14;23(8):3986. doi: 10.3390/s23083986 (PMC10141493; doi:10.3390/s23083986)
Supplement: Supplementary file 1 [file sensors-23-03986-s001.zip › sensors-2194849-supplementary.pdf]

Supplementary Materials

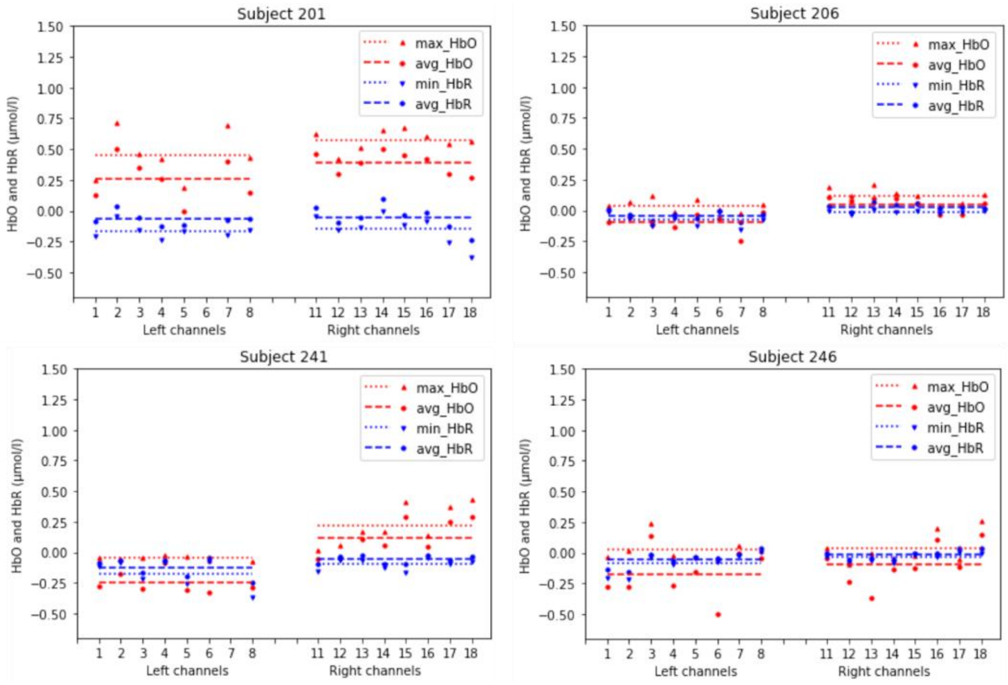

Figure S1. Detailed values of HbO (maximum and average) and HbR (minimum and average) for each channel on the left and right hemisphere for four persons. These plots show positive and negative values are visible for HbO and HbR, and the high variability depending on the channels.

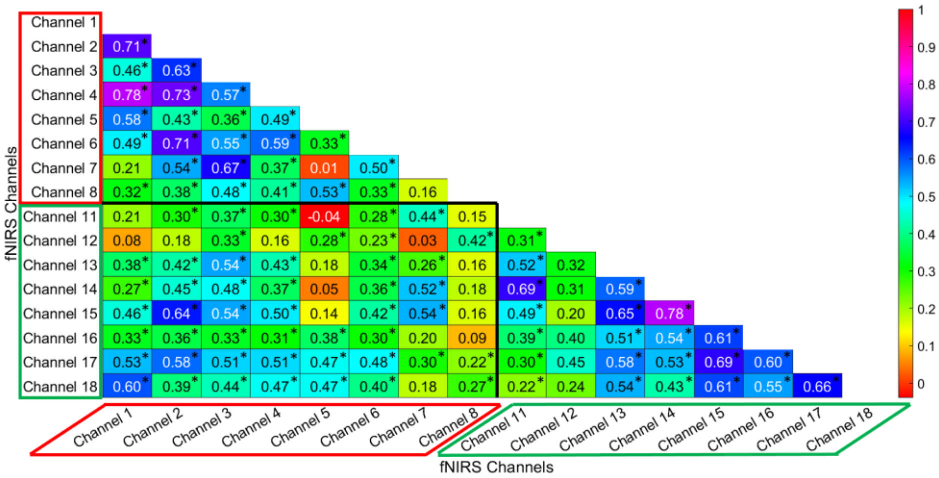

Figure S2. Correlations matrix representing the left (red) and right (green) channels in the axes. The black solid line traces the separation of the left and right channels. Significant correlations are marked with *.

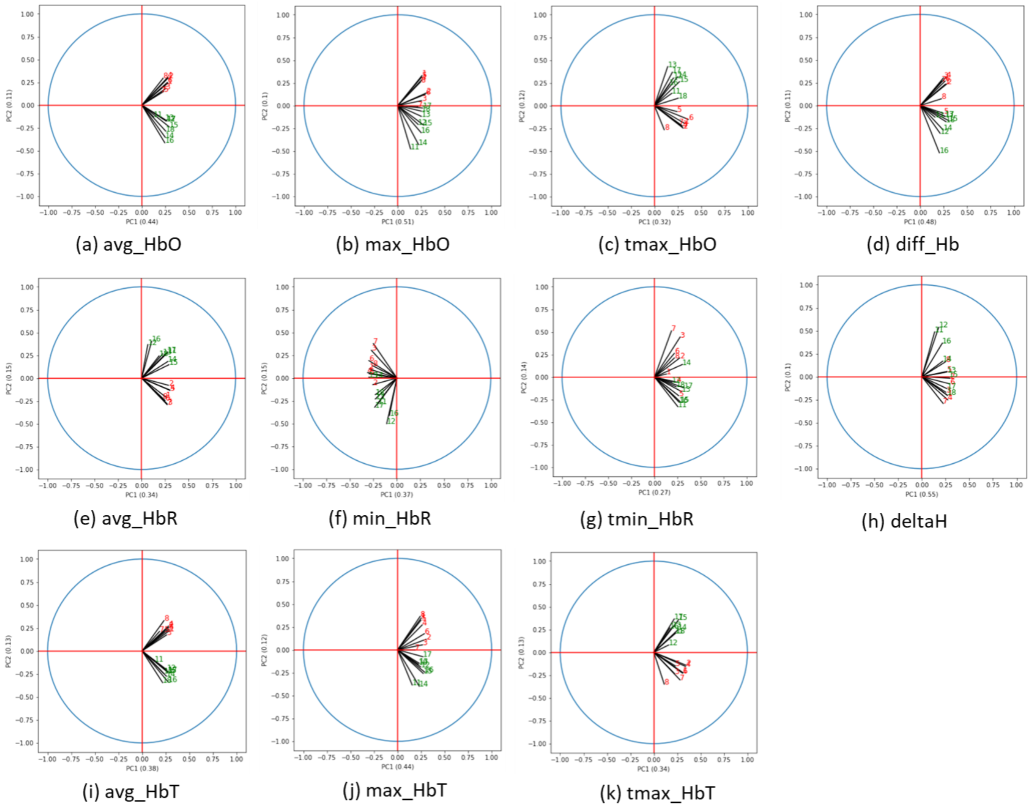

Figure S3. Representation of the first two components of the Principal Component Analysis of the left (red) and right (green) 16 channels used in fNIRS.

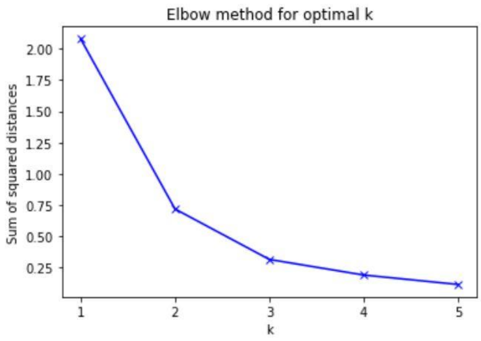

Figure S4. K-means elbow method to find the number of clusters that provides the best compromise to get less variations within clusters and clusters as different as possible. The inflection point is obtained when using two clusters.

An interesting use of clustering methods can be found in Phan et al. [71] where a combination of K-means and ensemble clustering methods are used to find how many lifting techniques can be found in people with chronic low back pain. The clusters are obtained from data extracted from video about the range of motion of knee, hip and trunk and four clusters were found and revealed four lifting techniques.
